# Variation in complex mating signals in an “island” hybrid zone between *Stenobothrus* grasshopper species

**DOI:** 10.1002/ece3.2265

**Published:** 2016-06-26

**Authors:** Jan Sradnick, Anja Klöpfel, Norbert Elsner, Varvara Vedenina

**Affiliations:** ^1^ Department of Neurobiology Johann‐Friedrich‐Blumenbach‐Institute Göttingen Germany; ^2^ Institute for Information Transmission Problems Russian Academy of Sciences Bolshoy Karetny per. 19 Moscow 127051 Russia; ^3^ Present address: Division of Nephrology Department of Internal Medicine III Dresden University of Technology Dresden Germany

**Keywords:** Courtship song, geometric morphometrics, hybrid zone, linkage disequilibrium, sexual selection, *Stenobothrus*

## Abstract

Two grasshopper species *Stenobothrus rubicundus* and *S. clavatus* were previously shown to meet in a narrow hybrid zone on Mount Tomaros in northern Greece. The species are remarkable for their complex courtship songs accompanied by conspicuous movements of antennae and wings. We analyzed variations in forewing morphology, antenna shape, and courtship song across the hybrid zone using a geographic information system, and we documented three contact zones on Mount Tomaros. All male traits and female wings show abrupt transitions across the contact zones, suggesting that these traits are driven by selection rather than by drift. Male clines in antennae are displaced toward *S. clavatus*, whereas all clines in wings are displaced toward *S. rubicundus*. We explain cline discordance as depending on sexual selection via female choice. The high covariance between wings and antennae found in the centers of all contact zones results from high levels of linkage disequilibria among the underlying loci, which in turn more likely results from assortative mating than from selection against hybrids. The covariance is found to be higher in *clavatus*‐like than *rubicundus*‐like populations, which implies asymmetric assortative mating in parental‐like sites of the hybrid zone and a movement of the hybrid zone in favor of *S. clavatus*.

## Introduction

Hybrid zones provide excellent models for the study of speciation because hybridizing taxa offer good experimental material for studying the evolution of reproductive barriers. Interspecific reproductive barriers can be classified into premating, postmating prezygotic, and postzygotic isolation mechanisms according to the time when they occur during the life cycle (Dobzhansky 1937; Mayr 1963; Coyne and Orr 2004). Numerous studies suggest that premating isolation evolves more quickly than postzygotic barriers, especially when premating isolation is achieved by assortative mating that evolved as a result of sexual selection (e.g., West‐Eberhard 1983; Andersson 1994; Panhuis et al. 2001).

Hybrid zones are often characterized by clinal changes in phenotypic traits and in allele frequencies that are maintained by a balance between gene flow and selection (Barton and Hewitt 1985; Barton and Gale 1993). Morphological and mating signals are likely to be exposed to both natural and sexual selection, although to different extents. Morphological features are usually not strongly sexually selected, whereas male calls or mating coloration is known to have a role in mate attraction and are therefore suggested to be more strongly subjected to sexual selection. For example, in the hybrid zone between the grasshoppers *Chorthippus brunneus* and *Ch. jacobsi* in northern Spain, the width of the cline for song characters is significantly narrower than for peg number, suggesting that mating signals may be associated with reduced hybrid fitness in the field (Bridle and Butlin 2002). On the other hand, in the Croatian hybrid zone between *Bombina* toad species, different quantitative traits show similar cline widths, even though they are likely to differ in their contribution to individual fitness (Nürnberger et al. 1995; Kruuk^1997^). This concordance in cline width for different quantitative traits may exist because high levels of linkage disequilibria are generated at the zone center by the contribution of many loci to hybrid unfitness (Szymura and Barton 1991).

**Figure 1:**
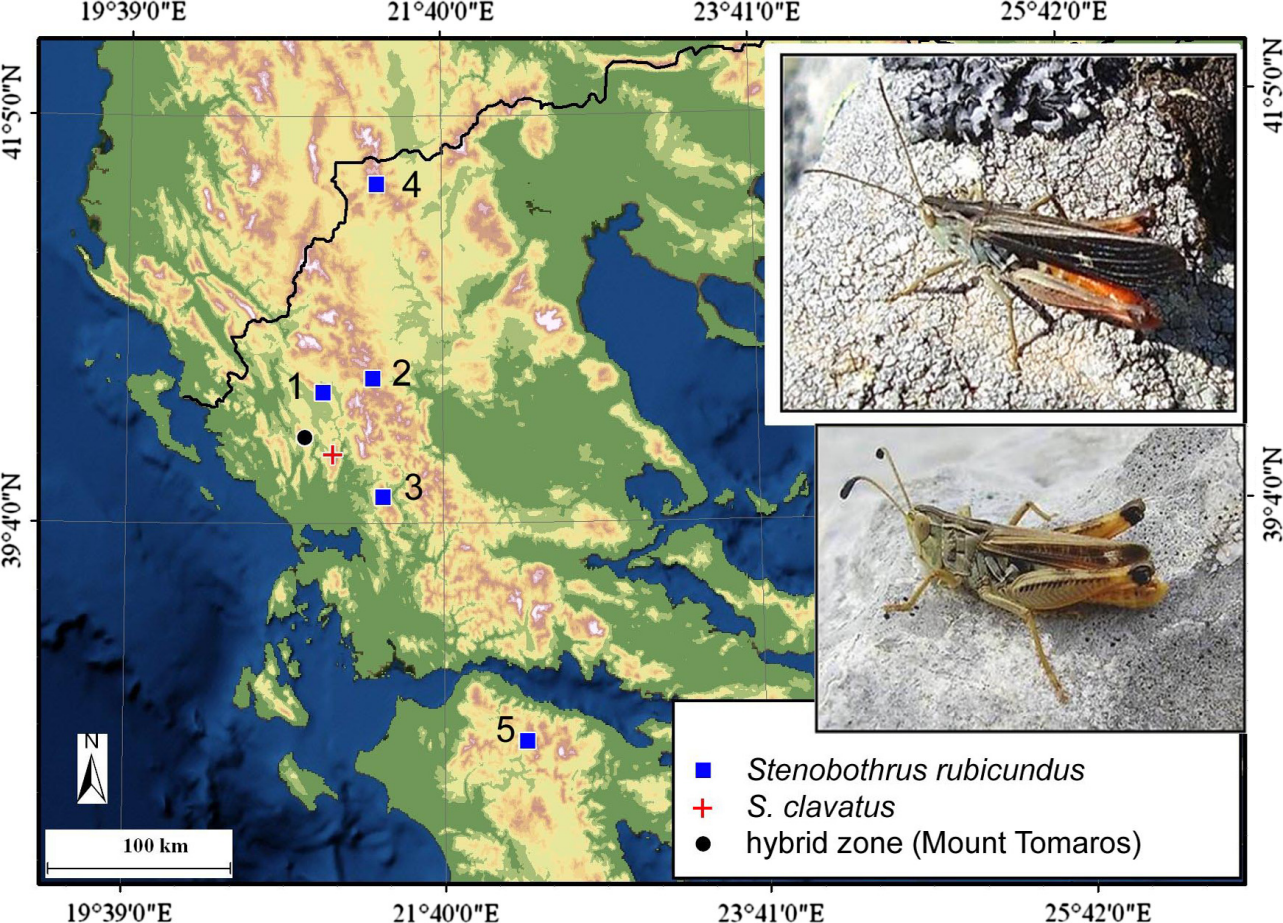
Locations of the allopatric populations of *Stenobothrus rubicundus* (1 – Mount Mitsikeli, 2 – Mount Valtou, 3 – Mount Pindos, 4 – Mount Vernon, 5 – Mount Aroania), the allopatric population of *S. clavatus,* and the hybrid zone in Greece. *S. rubicundus* is shown in the upper photograph; *S*. *clavatus* is shown in the lower photograph.

A recently discovered hybrid zone between the related grasshopper species *Stenobothrus rubicundus* and *S. clavatus* at Mount Tomaros in northern Greece is of special interest (Elsner et al. 2009; Vedenina et al. 2012, 2013). These species (Fig. 1) are remarkably different in several morphological characters (wings, antennae, stridulatory pegs), as well as in acoustic signals. During courtship behavior, these species not only produce a very complex sound signal but also use either antennae or wings for visual display. When courting, *S. rubicundus* males stridulate not only with the hind legs but also with the wings, and they alternate between these two mechanisms in a specific order (Elsner and Wasser 1995). *S. clavatus* males conspicuously move the antennae when producing a particular phase of courtship (Ostrowski et al. 2009). Thus, we expect that morphological features and mating signals in these hybridizing species may be similarly exposed to sexual selection.

The hybrid zone between *S. rubicundus* and *S. clavatus* is notable because the occurrence of both species on the Mount Tomaros is limited by elevation: they do not occur lower than 1300 m above sea levels (Elsner et al. 2009). The area of distribution of these species on Mount Tomaros covers not more than 16 km^2^. Thus, one can identify this hybrid zone as a “sky island” zone, in which such processes as the dispersal of parental genotypes into the contact zone, the extent of exchange of gene flow and the rate of hybridization arise in a special way. For example, the dispersal of parental genotypes into such a contact zone may be highly restricted or temporarily absent. The exchange of gene flow, by contrast, may be rather high. This may happen, first, because of the small area of distribution of the two species on Mount Tomaros and second, because of the preferences of parental phenotypes for different habitats that are patchily distributed over Mount Tomaros (Vedenina et al. 2012). Either factor could explain the hybrid zone's mosaic structure, which was identified based on courtship song analysis.

Courtship song analyses also showed that the hybrid zone between *S. rubicundus* and *S. clavatus* is narrow relative to dispersal (Vedenina et al. 2012). Narrow hybrid zones may be maintained by an equilibrium between dispersal, which increases zone width, and selection against hybrids, which reduces zone width (Barton and Hewitt 1985, 1989; Barton and Gale 1993). This model seems to only partly fit the hybrid zone between *S. rubicundus* and *S. clavatus*. We hypothesize that not only selection against hybrids but also assortative mating between parental phenotypes contribute to the reduction of the hybrid zone width, as it was shown in different hybrid zones between other grasshopper species (Bridle and Butlin 2002; Vedenina 2011). In experiments with playback of the courtship songs, females of *S. clavatus* and *S. rubicundus* preferred the songs of conspecific males. By contrast, hybrid females showed a lower selectivity than parental females, responding with approximately equal eagerness to playback of the parental and hybrid songs (Vedenina et al. 2013). These results suggest that hybrid males may lose in competition to males of parental species: hybrid males are mainly chosen by hybrid females, whereas parental males are attractive to all types of females. By contrast, hybrid females may even have an advantage over parental females because hybrid females may find a mate more quickly than the parental ones. Genetic incompatibility between the two species seems to be rather weak: the crosses between *S. rubicundus* females and *S. clavatus* males resulted in viable and fertile offspring, although the reciprocal crosses resulted in a smaller number of offspring, although these were still viable and fertile (Vedenina et al. 2012). Thus, assortative mating could contribute even more to the dynamic width of the hybrid zone than selection against hybrids.

This study has two major goals. First, we describe the structure of the hybrid zone on Mount Tomaros using a geographic information system (GIS). We examine how two morphological characters (forewing morphology and shape of antennae) and courtship song change across the hybrid zone. The integration of GIS into hybrid zone research began with Kohlmann et al. (1988) and was developed by Kidd and Ritchie (2000); see also Swenson (2008). Second, we study the strength of selection against hybrids and investigate which traits may be under stronger selection. This task is of special interest because all three phenotypic traits are presumably strongly affected by sexual selection. This goal is achieved, first, by an analysis of the cline width and position for three phenotypic traits across the hybrid zone, and second, by an analysis of the changes in variance and covariance between the traits across the hybrid zone. Covariance between traits is calculated separately for the allopatric sites and hybrid sites of three types: *clavatus*‐like, intermediate, and *rubicundus*‐like. This shows whether covariance is attributable to pleiotropy or linkage disequilibrium.

## Materials and Methods

### Specimens and localities

All specimens were collected over the summer seasons of 2005–2009 in various localities of Greece, at elevation not lower than 1300 m above sea level. Allopatric *S. rubicundus* was collected on Mount Mitsikeli (Ipiros, near the town of Ioannina, between 1300 m and 1810 m a.s.l.), Mount Valtou (Central Greece, 25 km E of the town of Arta, between 1758 m and 1781 m a.s.l.), Mount Pindos (Ipiros, 14 km E of the town of Metsobon, between 1720 and 1770 m a.s.l.), and Mount Vernon (Macedonia, Florina, between the town of Florina and Lake Prespa, between 1800 m and 2128 m a.s.l.) in northern Greece and on the Helmos peak of Mount Aroania (Akhaia, 20 km SE of Kalavrita village, between 1600 m and 2000 m a.s.l.) in Peloponnesus (Fig. 1). In contrast to *S*. *rubicundus* inhabiting the Alps, the Balkans, and the Southern Carpathians (Berger et al. 2010), *S. clavatus* is suggested to be a very local endemic of Greece; up to now, only two localities of *S. clavatus* are known there. Allopatric *S. clavatus* was only found on the Mount Xerovouni (Ipiros, near Ioannina town, between 1300 m and 1600 m a.s.l.). Mount Tomaros, where the two species meet and hybridize, is situated approximately 20 km S of Ioannina town, Ipiros. Mounts Mitsikeli, Valtou, and Xerovouni were the closest allopatric localities to Mount Tomaros (23 km northeast, 20 km southeast and 16 km southeast from Mount Tomaros, respectively).

For morphometric measurements, the collected specimens were placed in 96% alcohol; for the song recordings, the specimens were placed in cages (10 × 10 × 15 cm) and fed with fresh *Festuca* grass. Further measurements and recordings were carried out at the Institute for Zoology and Anthropology in Goettingen. Some recordings were conducted in Greece. All material was further deposited in the Museum of Natural Science of Berlin.

### Morphometric measurements of forewings

**Figure 2:**
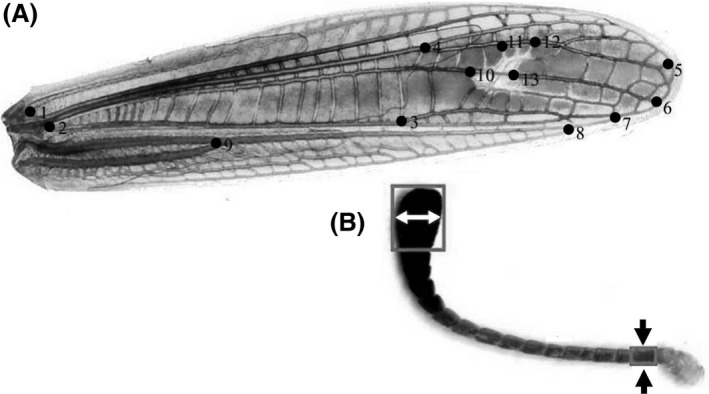
Forewing (A) and antenna (B) of *Stenobothrus clavatus*. (A) Description of landmarks: 1, division of costal and subcostal veins; 2, beginning of medial vein; 3, breaking of cubital vein; 4, first branching of radial vein; 5–8, endings of radial, medial, cubital, and anal veins, respectively; 9, fusion of anal veins; 10–13, landmarks indicating the location of the stigma. (B) The arrows indicate the width of the first and the last antenna segments.

To compare wing morphology in allopatric *S. clavatus,* allopatric *S. rubicundus*, and specimens from Mt. Tomaros, we use landmark‐based geometric morphometrics. For the forewing analysis, we selected 13 landmarks (Fig. 2A, description of landmarks is given in the figure legend). Wing size and shape variation was observed from 3308 specimens (195 males and 137 females of *S. rubicundus*, 131 males and 128 females of *S. clavatus*, 1631 males and 1086 females from Mount Tomaros). The right forewing of each specimen was mounted in glycerin between two cover glasses (24 × 60 mm), and digital images were captured with a scanner (Epson PERFECTION 4490 Photo) with a high resolution (4800 dpi; 3684 × 1644 pixels). Thirteen landmarks were digitized with TpsDig 2.0 software and expressed as x‐y coordinates in Cartesian space (Rohlf 2004). Direct analysis of the coordinates would be inappropriate as the effects of variation in orientation and sizes of the wings can introduce bias. This variation was mathematically removed using generalized procrustes analysis (Rohlf and Slice 1990) in CoordGen6 (Sheets 2000). Procrustes analysis superimposes landmark configurations of various wings using least‐squares estimates for translation and rotation. Transformed landmarks were used in subsequent principal component analysis (PCA) combined with canonical variate analysis (CVA) using the programs PCAgen6 and CVAgen6, respectively, to examine the pattern of among‐ and within‐species/population variation. CVA assesses the ability to assign specimens in a data set to groups (considering GPS data), rather than asking whether the groups are different. Additionally, squared Mahalanobis distances were derived from CVA in order to quantify interspecific phenetic relationships. To calculate the covariance between the wing and antenna traits, we further used the logarithmic values. Because the Mahalanobis distances varied between 0.01 and 3.9 in allopatric *clavatus* populations and between 19 and 25 in *rubicundus* populations, variances of the logarithmic values appeared to be much higher in *clavatus* than *rubicundus* populations. We also used the Mahalanobis distances to calculate two hybrid indices (HI). One hybrid index score was generated in the following way: scores 1–2 were given to allopatric *S. clavatus*, scores 11–12 were given to allopatric *S. rubicundus*, and scores from 3 to 10 were given to individuals with intermediate characters. This hybrid index was then standardized to vary from 0.0 to 1.0. The ranges of variation from 0 to 0.025 and from 0.975 to 1 corresponded to 1 and 12, respectively; the ranges from 0.025 to 0.1 and from 0.9 to 0.975 corresponded to 2 and 11, respectively; the range from 0.1 to 0.9 was divided by eight, with the other scores, 3–10, equally distributed along this range (Table 1).

**Table 1:** Two hybrid index scores used for analyses of the three phenotypic traits in the hybrid zone on Mount Tomaros. 1–2: *Stenobothrus clavatus*, 11–12: *S. rubicundus*

Hybrid index	Standardized hybrid index
1	0–0.025
2	0.025–0.1
3	0.1–0.2
4	0.2–0.3
5	0.3–0.4
6	0.4–0.5
7	0.5–0.6
8	0.6–0.7
9	0.7–0.8
10	0.8–0.9
11	0.9–0.975
12	0.975–1

### Morphometric measurements of antennae

Antennae were studied from 1047 specimens (36 males and 10 females of *S*. *clavatus*, 43 males and 24 females of *S. rubicundus*, 616 males and 318 females from Mount Tomaros). Digitized images of antennae were obtained by a method similar to that used for the wings: each antenna was mounted in glycerin between two cover glasses, and digital images were captured with a scanner with a resolution of 4800 dpi. Because the *S. clavatus* antennae are spatulate, we measured the width of the first and the last antenna segments using ImageJ software and then calculated the relative width of these two parameters (Fig. 2B). The hybrid indices were calculated the same way as for the wings.

### Courtship song analysis

Courtship song recordings were made in the laboratory from 175 males (12 males of allopatric *S*. *rubicundus*, 13 males of allopatric *S. clavatus,* and 150 males from Mount Tomaros). The low number of the males recorded in comparison with the number of the males studied morphologically is explained by the difficulty, in terms of both complexity and time requirement, of recording courtship behavior. In addition, all three traits were not always measured in the same specimen because forewings of some males could be slightly ragged after courtship recordings. This prevented the combination of a qualitative morphometric procedure and song analysis.

To record the courtship song, a male was placed near a female on a round, rotatable, and temperature‐controlled heating plate. The ambient temperature near a singing male varied between 35°C and 41°C. Not only the sound but also the movements of the hind legs during stridulation were recorded with an optoelectronic device (von Helversen and Elsner 1977). This technique allowed us to conduct a more comprehensive analysis of the elaborate song pattern. A small piece of light‐reflecting foil was attached to the distal end of each hind femur. A light spot sent through the camera's optics illuminated the foil and generated a voltage in a photosensor that was linearly correlated to the position of the light spot on the hind leg. Stridulatory sounds were recorded with a piezo microphone connected to a flexible rod. All data were digitally stored on a computer via a data acquisition card (National Instruments, Austin, TX) with the software LabVIEW 7 (National Instruments) and visualized later with DIADEM 9.1 (National Instruments) and TurboLab 4.0 software (Bressner Technology, Germany). The sampling rate was 40 kHz for recording both sound and stridulatory movements.

Courtship songs of *S. clavatus* and *S. rubicundus* have previously been described in detail by Elsner and Wasser (1995) and Ostrowski et al. (2009). Courtship songs of hybrids were described by Vedenina et al. (2012). Based on the song analysis, we selected eight song characters and applied the PCA to all of them. The values of the first principal component (PC1) were used for the calculation the hybrid indices, which were estimated in the same way as for the wings and antennae.

### Geographic Information Systems (GIS) analysis

For all 156 sites studied on Mount Tomaros, we determined their latitude, longitude, and elevation (Table S1). The number of specimens collected in each site varied from 2 to 110 (20 specimens on average), each site had a radius of approximately 20 m, and the sites were typically between 50 m and 500 m apart. To interpolate trait values at unsampled sites, we used a surface‐generation algorithm described by Kidd and Ritchie (2000). In this distance‐based method (Inverse Distance Weighting, IDW), the closer the predicted grid cell is to a known population in Euclidean distance, the more similar the predicted value for that cell is to that of the known populations. Therefore, a grid cell lying precisely halfway two known populations with different trait values will have the average value of those two populations (Swenson 2008). A grid of cells of 50 m × 50 m in size was interpolated both for morphological traits and for the courtship song using the program ArcGIS (ESRI, Redlands, CA). The program used the following formula:

$$V=\frac{\sum_{i=1}^{n}v_{i}\frac{1}{d_{i}^{p}}}{\sum_{i=1}^{n}\frac{1}{d_{i}^{p}}}$$

Here, *v*_*i*_ represents the nearest sample sites, and *d*_*i*_ is the Euclidean distance from *v*_*i*_ to the distance‐weight cell. The formula describes exponential distances ($d_{i}^{p}d_{n}^{p}$ ) from the known points (*n*) to unknown points (Ware et al. 1991). After the continuous trait surface was created, the cells with values 0.49 < *P* < 0.51 formed an almost continuous curve, from which the center of the hybrid zone was estimated. We defined the distance to the cline center as the shortest distance between each site and that curve, thus reducing the data to a one‐dimensional transect.

### Cline analysis

To illustrate a degree of similarity of clines in different characters, the clines were fitted with a Loess smoothing function and superimposed. To compare width and placement of different clines, the regression methodology suggested by Barton and Hewitt (1985) and Kruuk (1997) was used. If the clines in two characters have identical widths and central placements, regressing one character on another will give a straight line; if a cline in one character is displaced in space relative to another, then the quadratic term will be significant; if a cline in one character is narrower or wider than in another, the cubic term will be significant. This indirect approach allows the avoidance of problems associated with any spatial analysis, such as when one should measure the geographic distances across a mosaic hybrid zone.

### Estimating variance and covariance between different characters

Phenotypic variance is expected to highly increase in the center of a hybrid zone when a trait exhibits a bimodal distribution. To compare changes in variances across the hybrid zone between *S. clavatus* and *S. rubicundus*, only localities with sample sizes of greater than five were used. Measures of linkage disequilibrium (LD) may be indicative of the strength of selection against hybrids and serve as a sign of whether the introgression occurs close to the center of the hybrid zone. LD can be estimated from covariance between two quantitative traits, assuming that two traits, *z* and *z*΄, are influenced by the additive effects of separate sets of genes and that populations do not differ significantly from Hardy–Weinberg equilibrium (Nürnberger et al. 1995):

$$D^{*}=\frac{2\text{cov}(z,z^{\prime })}{\Delta z\Delta z^{\prime }}$$

At the same time, covariance between phenotypic traits may result either from LD or pleiotropy. This assumption is evaluated by calculating the covariance between the traits in allopatric and hybrid populations. In this case, it is important that variance in allopatric populations is not equal to zero, which means that relevant loci with pleiotropic effects are polymorphic in these populations.

## Results

### Variation in forewing morphology across the hybrid zone

**Figure 3:**
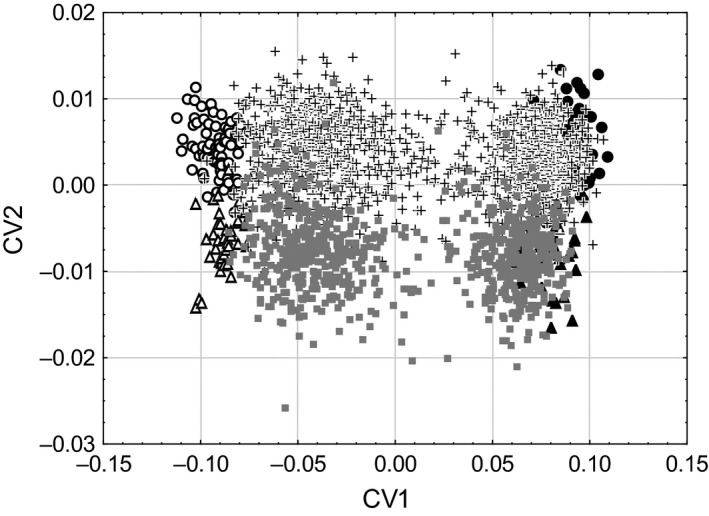
Plot of the first two canonical variables (CV1 and CV2) illustrating the differences in the forewing morphology between allopatric *Stenobothrus clavatus* (open marks) and allopatric *S. rubicundus* (black marks); the differences are similar in males (circles) and females (triangles). Specimens from Mount Tomaros (crosses in males and squares in females) are split into two groups: *rubicundus*‐like and *clavatus*‐like specimens.

We did not find significant differences in the wing morphology among five allopatric populations of *S. rubicundus* (CVA/MANOVA test, *P *>* *0.5). At the same time, the difference between allopatric *S*. *rubicundus* and *S. clavatus* was highly significant both in males and in females (CVA/MANOVA test, *P *<* *0.001). The results of CVA applied to allopatric populations and specimens from Mount Tomaros also show the difference between allopatric specimens (Fig. 3). Comparison of CV1 scores shows that specimens from Mount Tomaros could be split into two groups: *rubicundus*‐like and *clavatus*‐like specimens. To some degree, the specimens from Mount Tomaros overlapped with allopatric specimens. Most of them, however, were different from allopatric specimens. Intermediate phenotypes were in the minority. Notably, a bimodal distribution of CV1 scores was found in both sexes.

**Figure 4:**
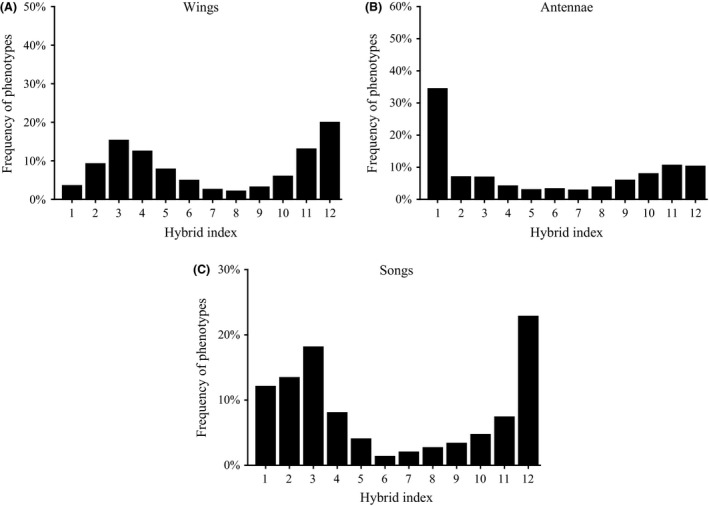
The frequency distribution of the hybrid index scores on Mount Tomaros shows a variety of wing phenotypes (A), antenna phenotypes (B), and courtship song phenotypes (C). “1” corresponds to *Stenobothrus clavatus* and “12” to *S. rubicundus*.

The overall frequency distribution of the HI scores on Mount Tomaros shows a variety of wing phenotypes (Fig. 4A). However, the distribution was bimodal and asymmetrical: among the *rubicundus*‐like phenotypes, individuals with extreme HI (as in allopatric populations) dominated, whereas among the *clavatus*‐like phenotypes, individuals with extreme HI typical for allopatric *S. clavatus* were in the minority.

**Figure 5:**
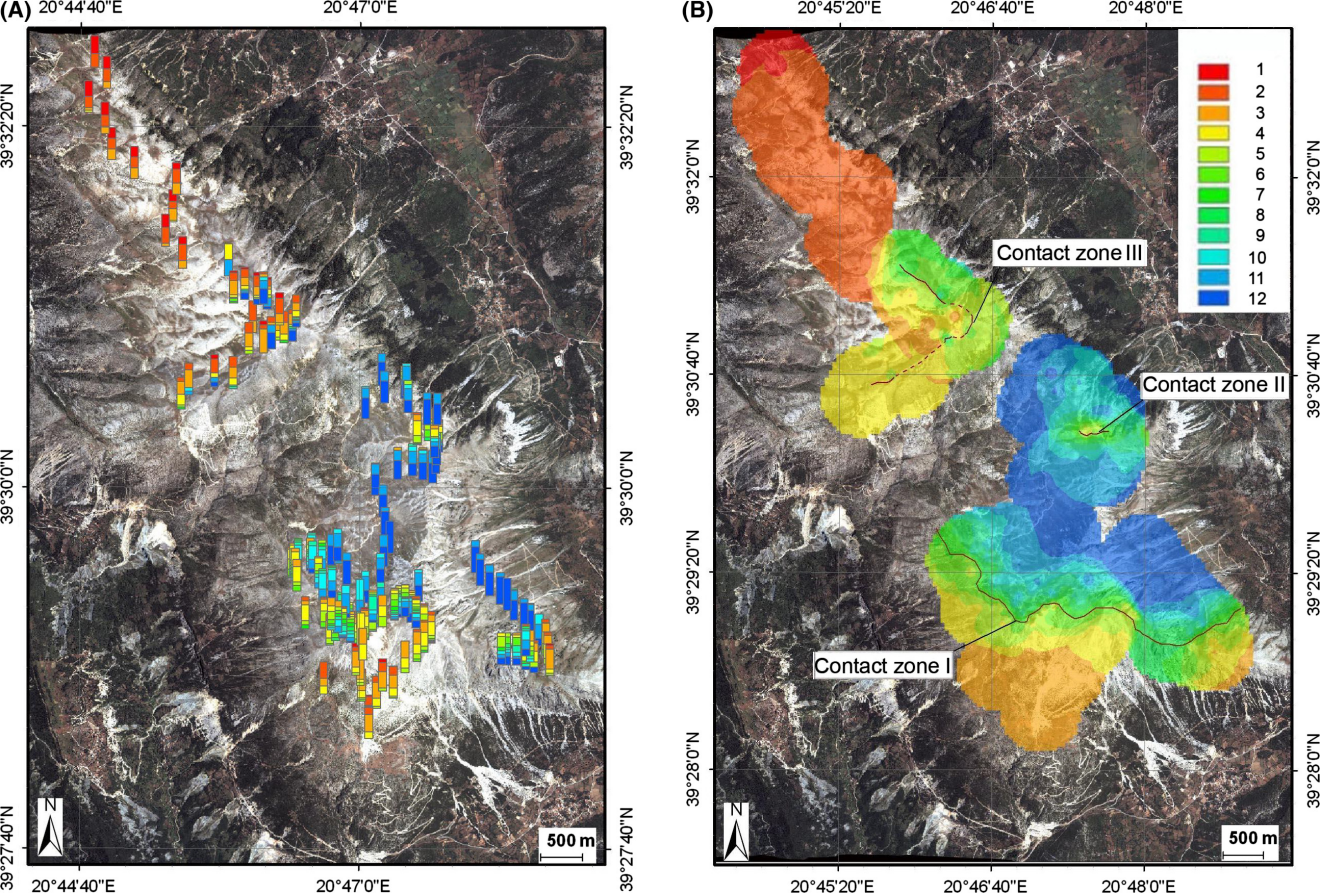
Maps of Mount Tomaros with sampling locations of wing phenotypes (A) and with interpolated wing trait values at unsampled sites (B). Hybrid indices from 1 (*S. clavatus*) to 12 (*S. rubicundus*) indicated by different colors are shown at the right. Centers of the three contact zones are shown by the black lines.

**Figure 6:**
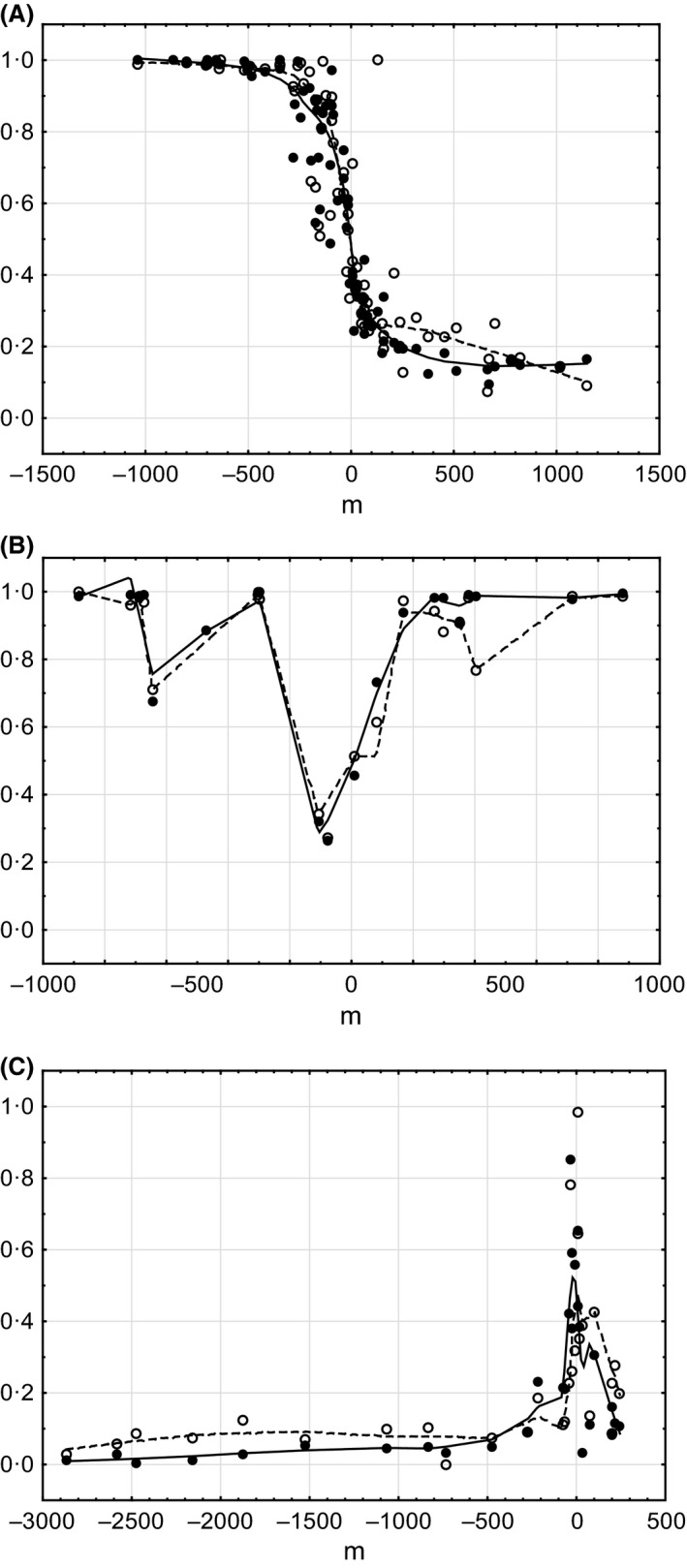
Transitions in the forewing morphology across the three contact zones (A, contact zone I; B, contact zone II; C, contact zone III) in males (black symbols) and females (white symbols). Each point is a locality mean. *y* coordinate – standardized hybrid index. Each curve (solid for males and dashed for females) is fitted with a Loess smoothing function.

The distribution of the wing phenotypes in different sites of Mount Tomaros, as shown in Figure 5A, reveals a complex pattern. The sites with the *clavatus*‐like populations were found in the northern and southern parts of Mount Tomaros; however, the extreme parental phenotypes occurred more often in the northern than in the southern sites. In the northeastern part of Mount Tomaros, mainly *rubicundus*‐like populations were found, and only a few sites appeared to be intermediate between *S. rubicundus* and *S. clavatus*. To understand such a complex pattern of spatial distribution of phenotypes, we distinguished three contact zones on Mount Tomaros (Fig. 5B) and analyzed the variation in trait means separately across the three transects and separately for males and females (Fig. 6). The clines for males and females along the southern transect (contact zone I, CZI) have clearly sigmoid shapes. The northern tails of the clines reach the values of allopatric *S. rubicundus*; the southern tails, however, do not reach the values of allopatric *S. clavatus* (Fig. 6A). The clines along the northeastern transect (contact zone II, CZII) are clearly not sigmoid: the means correspond to the values of allopatric *S. rubicundus* at the northern ends; the clines then fall around the centers, although not reaching the values of allopatric *S. clavatus*, and rise again at the southern ends (Fig. 6B). In the northern transect (contact zone III, CZIII), the clines are somewhat similar to those found in CZII: the northern tails of the clines reach the values of allopatric *S. clavatus*, the clines then rise to the values of allopatric *S. rubicundus* around the centers, and they then fall at the southeastern end (Fig. 6C). The distribution of means in CZI was clearly bimodal for both sexes. By contrast, the distribution of means in CZII and CZIII for both sexes was unimodal, with predomination of *S. rubicundus* phenotypes in CZII and *S. clavatus* phenotypes in CZIII. In all transects, variations of means correlated strongly between males and females (Spearman's rank‐order correlation, *r* = 0.74–0.9, *P *<* *0.001).

### Variation in antenna morphology across the hybrid zone

**Figure 7:**
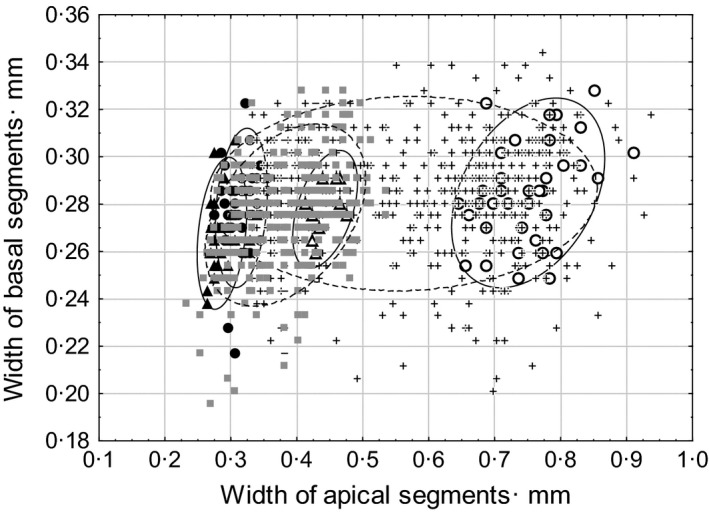
Plots of widths of apical (*x*‐axis) and basal (*y*‐axis) antenna segments in males and females illustrating large differences between males of allopatric *Stenobothrus clavatus* (open circles) and males of allopatric *S. rubicundus* (black circles), and the much smaller differences between females of allopatric *Stenobothrus clavatus* (open triangles) and females of allopatric *S. rubicundus* (black triangles). Males from Mount Tomaros (crosses) are split into two groups, *rubicundus*‐like and *clavatus*‐like specimens, whereas females from Mount Tomaros (squares) form a homogenous group.

Differences in the antenna morphology between allopatric populations of *S. rubicundus* and *S. clavatus* were highly significant for both males (*t*‐test: *t*‐value = 43.2, df = 77, *P *<* *0.001) and females (*t*‐value = 18.5, df = 30, *P *<* *0.001). Comparison of allopatric populations and populations from Mount Tomaros showed that some specimens from Mount Tomaros overlapped with allopatric specimens, but most individuals differed from allopatric specimens (Fig. 7). All male individuals could be split into two groups: *rubicundus*‐like and *clavatus*‐like specimens (Fig. 7A), similarly to the distribution found for the male wings. By contrast, the distribution frequency of antenna phenotypes in females was much more homogenous, that is, parental‐like phenotypes occurred approximately as often as intermediate ones (Fig. 7B), unlike the distribution found for the female wings.

The overall frequency distribution of the HI scores for the antenna morphology in the hybrid zone was slightly different from that found for the wings (Fig. 4B). The pure *clavatus* antenna phenotype clearly dominated, and bimodality was not as obvious in antennae as in the wing phenotype distribution. Intermediate indices for the antenna morphology were more uniformly distributed than those for the wing morphology.

**Figure 8:**
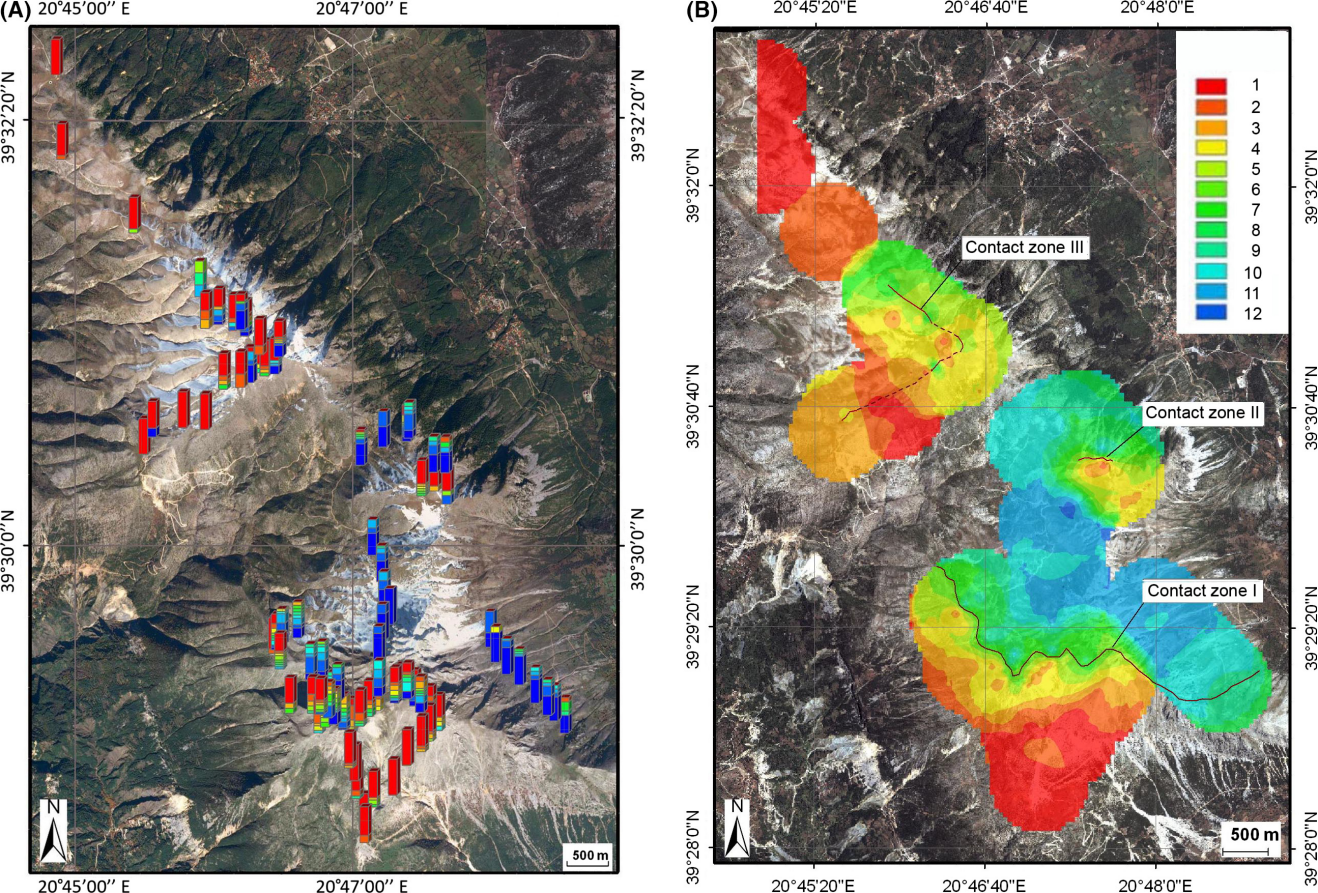
Maps of Mount Tomaros with sampling locations of antenna phenotypes (A) and with interpolated antenna trait values at unsampled sites (B). Hybrid indices from 1 (*S. clavatus*) to 12 (*S. rubicundus*) indicated by different colors are shown at the right. Centers of the three contact zones are shown by the black lines.

**Figure 9:**
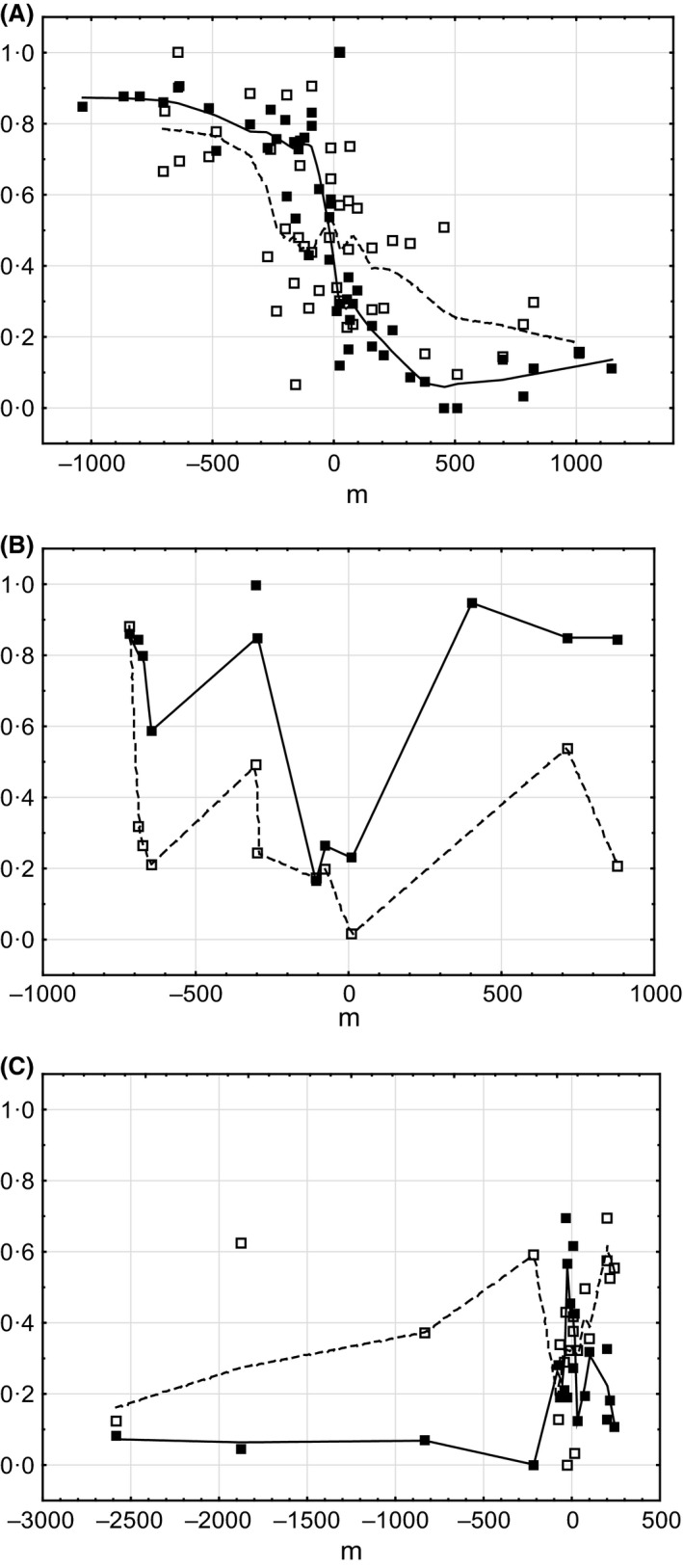
Transitions in the antenna morphology across the three contact zones (A, contact zone I; B, contact zone II; C, contact zone III) in males (black symbols) and females (white symbols). Each point is a locality mean. *y* coordinate – standardized hybrid index. Each curve (solid for males and dashed for females) is fitted with a Loess smoothing function.

Analysis of the spatial distribution of the antenna phenotypes in the hybrid zone allowed us to distinguish three contact zones (Fig. 8), similar to our observations for the wing phenotypes. However, in contrast to the wing phenotype distribution, the extreme *clavatus* antenna phenotypes seem to occur equally often in the northern and southern parts of Mount Tomaros. The shapes of the antenna clines along the three transects are similar to the shapes of the wing clines in males, but this is not the case in females (Fig. 9). The male cline across CZI has a sigmoid shape; by contrast, the female cline has a much shallower slope (Fig. 9A). Moreover, the distribution of means across the contact zone was bimodal for males and unimodal for females, which corresponded to the normal distribution (Kolmogorov–Smirnov test, *d* = 0.09, *P *>* *0.2). Meanwhile, the correlation between antenna means in males and females was significant (Spearman's rank‐order correlation, *r* = 0.6, *P *<* *0.05). In CZII, the male cline corresponds to the values of allopatric *S. rubicundus* at the northern ends; it then falls around the center, almost reaching the values of allopatric *S. clavatus* and then rises again at the southern end (Fig. 9B). In CZIII, the northern tail of the male cline reaches the values of allopatric *S. clavatus*; the cline rises around the center, although not reaching the values of allopatric *S. rubicundus,* and it then falls again at the southeastern end (Fig. 9C). Thus, the distribution of the male phenotypes across CZII and CZIII was unimodal and similar to that found for the wing phenotypes, with dominating *S. rubicundus* phenotypes in CZII and of *S. clavatus* phenotypes in CZIII. By contrast, the female clines across CZII and CZIII have more complex shapes than the male clines, without any clear pattern. In both zones, the distribution of the female phenotypes corresponded to the normal distribution (*d* = 0.14–0.23, *P *>* *0.2). We did not find any significant correlation between male and female antennae along transects II and III.

### Variation of courtship songs across the hybrid zone

The overall frequency distribution of HI scores for courtship song in the hybrid zone was typically bimodal (Fig. 4C). Most individuals from Mount Tomaros could be separated into *clavatus*‐like and *rubicundus*‐like specimens, whereas intermediate phenotypes were rare. The distribution resembles that found for the wing phenotypes: individuals with extreme HI dominated among the *rubicundus*‐like phenotypes but not among the *clavatus*‐like phenotypes.

**Figure 10:**
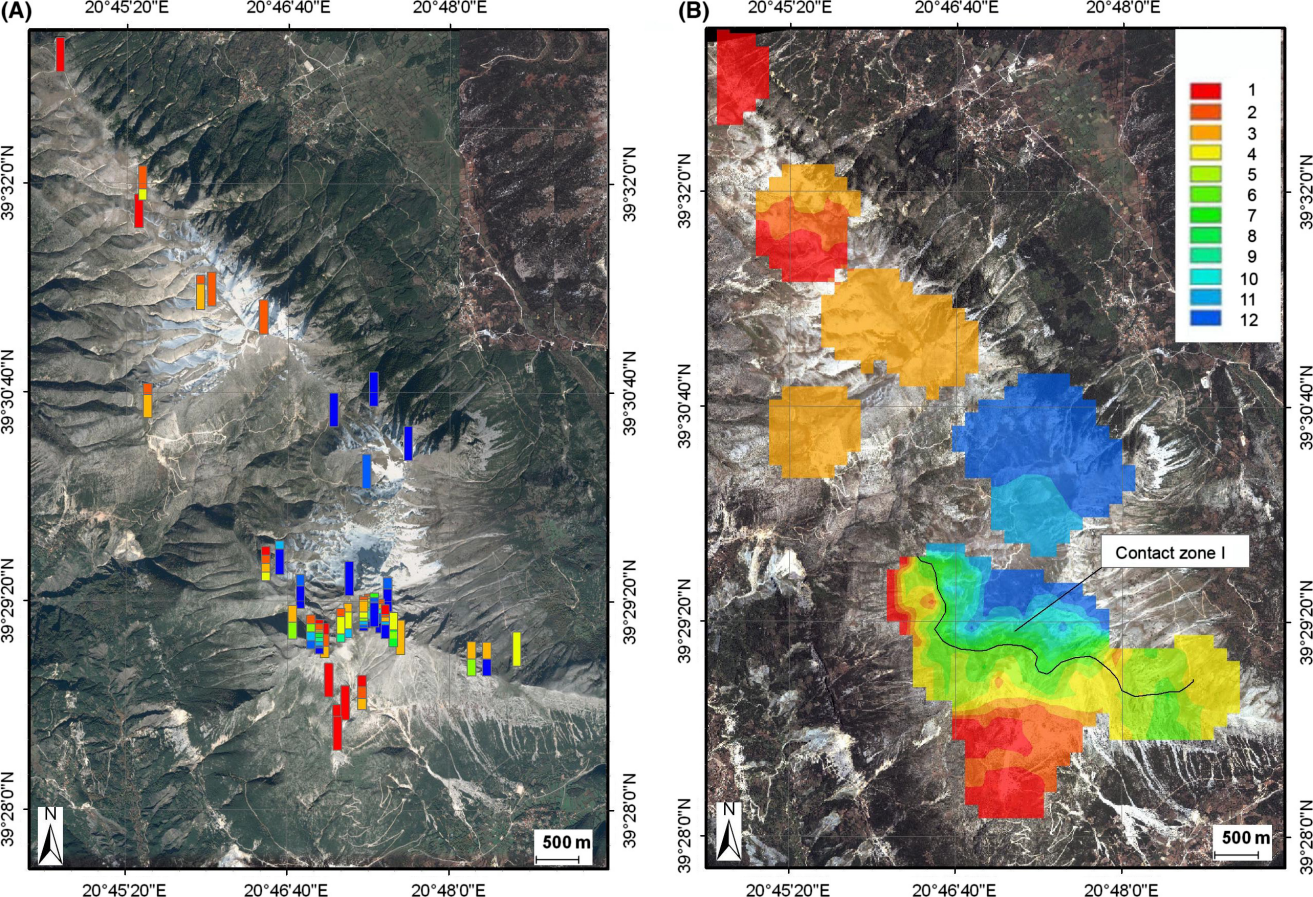
Maps of Mount Tomaros with sampling locations of courtship song phenotypes (A) and with interpolated song trait values at unsampled sites (B). Hybrid indices from 1 (*S. clavatus*) to 12 (*S. rubicundus*) indicated by different colors are shown at the right. The center of contact zone I is shown by the black line.

**Figure 11:**
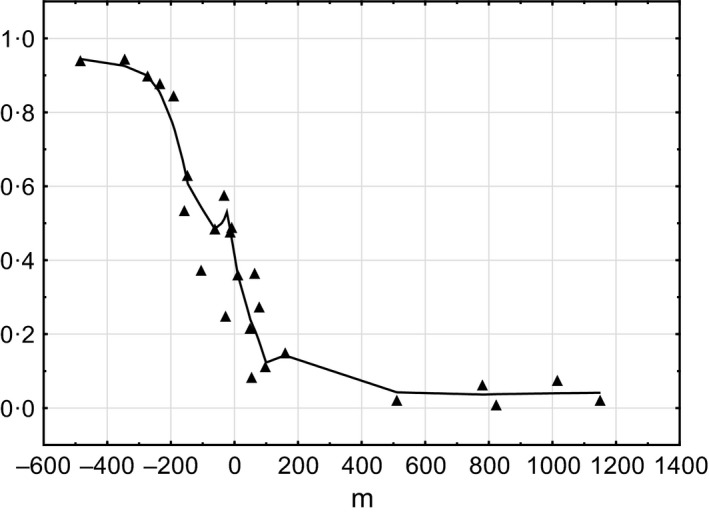
Transition in the courtship song across the contact zone I. Each point is a locality mean. *y* coordinate – standardized hybrid index. The curve is fit with a Loess smoothing function.

The spatial distribution of the courtship song phenotypes in the hybrid zone showed only one contact zone, in the southern part of Mount Tomaros (CZI). This may be explained by the lower number of sites for which songs were recorded (Fig. 10A), compared to the sites for which the wings and the antennae were studied. In the northern part of Mount Tomaros, all song phenotypes were found to be *clavatus*‐like, whereas in the northeastern part of Mount Tomaros, they were of the *rubicundus* type. The distribution of the song phenotypes in CZI was somewhat similar to that obtained for the antenna morphology, with pure *clavatus* phenotypes found at the southern end of the contact zone (Fig. 10B). Meanwhile, *clavatus*‐like song phenotypes were also found in the most eastern sites of the contact zone, which was similar to the wing pattern distribution. The song cline across CZI has a sigmoid shape (Fig. 11) similar to those found for the wings and the male antennae.

### Comparison of clines in different characters

To compare central placement and width of the clines along all three contact zones of Mount Tomaros, transect‐independent regression was used (Barton and Hewitt 1985; Kruuk 1997). Regressions for the three characters against the summarized hybrid index are presented in Table 2. In CZI, quadratic and cubic terms were found to be significant for male and female antennae and for female wings. In two other contact zones, by contrast, neither quadratic, nor cubic models approached significance. This could indicate the presence of different widths and positions of clines for male and female antennae and for female wings in CZI but not in other contact zones. Meanwhile, the values of coefficients (both quadratic and cubic) for female antennae appeared to be very large in all contact zones. Moreover, quadratic and cubic terms for all characters except for female antennae were consistent in sign (Table 2). This indicates a tendency for different widths and positions of the antenna and wing clines in all contact zones.

**Table 2:** Statistics for the regression analysis of the three characters studied in the *clavatus/rubicundus* hybrid zone against the mean hybrid index

Characters	Males				Females			
	*b* ^2^	*P*	*b* ^3^	*P*	*b* ^2^	*P*	*b* ^3^	*P*
Contact zone I								
Wings	0.99	0.39	−0.58	0.39	2.48	**0.028**	−1.50	**0.028**
Antennae	3.16	**0.015**	−2.13	**0.006**	−9.46	**0.012**	5.73	**0.010**
Songs	2.06	0.12	−1.13	0.14				
Contact zone II								
Wings	2.77	0.57	−2.44	0.36	0.26	0.98	−0.97	0.83
Antennae	5.32	0.28	−3.18	0.22	−15.3	0.17	10.3	0.09
Contact zone III								
Wings	1.79	0.34	−0.96	0.44	2.28	0.17	−1.16	0.30
Antennae	2.58	0.48	−1.43	0.53	−9.93	0.24	5.77	0.28

Significant levels of *b* ^2^ and *b* ^3^ terms are marked in bold.

**Figure 12:**
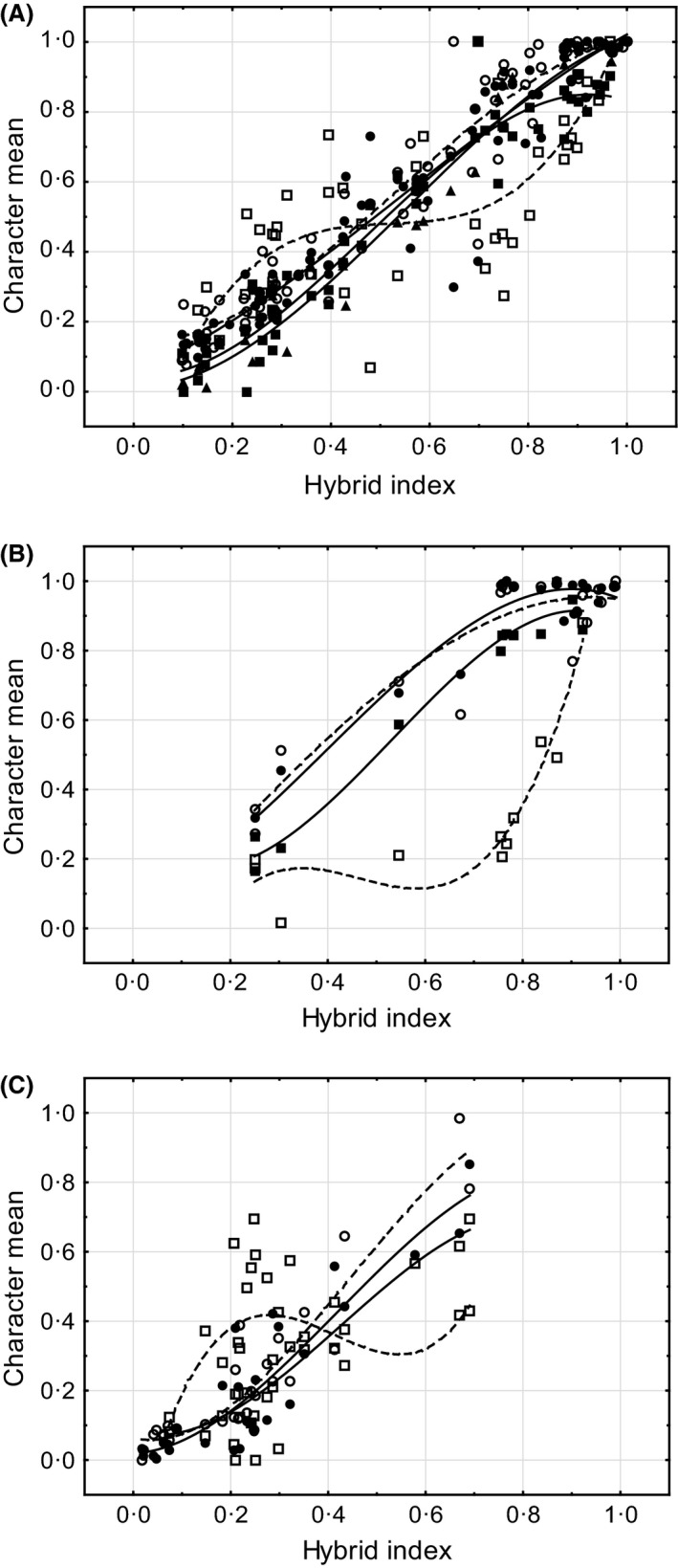
Character means (*y*‐axis) regressed on the mean hybrid index (*x*‐axis) for three contact zones (A, contact zone I; B, contact zone II; C, contact zone III). Circles indicate wing phenotypes, squares – antenna phenotypes, triangles – courtship song phenotypes. Males are indicated by black symbols, females – by white symbols. The lines represent the best‐fitting polynomial (of cubic order) equations. Regression coefficients and *P* values for these data are presented in Table 2.

The plot of each character against the hybrid index illustrates the use of this regression method to uncover differences in width and placement of the clines for different characters (Fig. 12). The differences between the curves for most of characters are small. Only the curves for female antennae behave differently in all three contact zones.

This regression analysis shows that wing clines are slightly wider than antenna clines in all contact zones, and wing clines are displaced more to *rubicundus* ends, whereas antenna clines are displaced to *clavatus* ends in every zone. All these clines are relatively narrow. This, however, is not the case for female antennae: in all contact zones, this character shows the widest clines, which implies very weak or absent pressure of selection on this character. In CZI, the quadratic and cubic terms did not differ significantly between male wings and songs, indicating coincident and concordant clines for these characters. However, considering the values of the coefficients, the song cline tends to be wider than the antenna cline but narrower than the wing cline; its central placement is between the placements of the antenna and wing clines.

### Covariances and estimates of linkage disequilibrium

Linkage disequilibria in hybrid populations should cause an increase in the covariance among quantitative traits in the center of the zone. We only computed the covariance among wings and antennae; statistics for the covariance between morphological and song characters was very low because these characters were not always studied in the same specimens (see Methods). The sampling sites were divided into five classes: allopatric *S. clavatus*, allopatric *S. rubicundus*, and three classes of sites from Mount Tomaros (with *clavatus*‐like phenotypes, *rubicundus*‐like phenotypes, and intermediate phenotypes).

To calculate the covariance, we used the logarithmic values, which decreased the difference between the values for wings and antennae (Table 3). Because of using the logarithmic values (see Methods), the variances for wings were found to be the highest in allopatric *clavatus* populations. Among the sites from Mount Tomaros, the highest variances for both morphological characters were found in the sites with intermediate phenotypes, that is, in the centers of each of three contact zones. Similar results were obtained for the covariance between the morphological traits: the highest covariance was found in the sites with intermediate phenotypes. At the same time, high and significant correlation between wings and antennae was only found in the centers of all three contact zones for males (Spearman's rank‐order correlation, −0.77 < *r* < −0.81, *P *<* *0.05), and not for females. The *clavatus*‐like populations showed higher covariance than *rubicundus*‐like populations, with greater covariance in males than in females (the correlation was, however, nonsignificant in both sexes).

**Table 3:** Estimates of variance, covariance, and linkage disequilibria (LD) for two morphological characters in allopatric populations of *S. clavatus* and *S. rubicundus* and in three contact zones of Mount Tomaros

		Males		Females	
		Wings	Antennae	Wings	Antennae
Δ*z*		7.72	1.22	7.43	0.756
Wings	allop. *clavatus*	*1.05* (94)	0.004 (36)	*1.14* (106)	−0.011 (9)
	Contact zone I				
	*clavatus*‐like	*0.16* (344)	−0.022 (152)	*0.15* (215)	0.002 (80)
	Intermediate	*0.33* (578)	−0.097* (157)	*0.24* (156)	−0.034 (56)
	*rubicundus*‐like	*0.01* (236)	−0.004 (103)	*0.008* (186)	−0.001 (70)
	Contact zone II				
	*clavatus*‐like	*0.084* (17)	−0.015 (14)	*0.15* (23)	0.007 (8)
	Intermediate	*0.32* (73)	−0.16* (19)	*0.43* (29)	−0.011 (7)
	*rubicundus*‐like	*0.004* (146)	−0.0003 (46)	*0.004* (65)	−0.0004 (24)
	Contact zone III				
	*clavatus*‐like	*0.49* (173)	−0.025 (58)	*0.55* (87)	−0.003 (32)
	Intermediate	*0.78* (82)	−0.29* (47)	*0.66* (66)	−0.066 (29)
	allop. *rubicundus*	*0.003* (168)	−0.001 (43)	*0.003* (124)	−0.002 (24)
Antennae	allop. *clavatus*	**0.0005**	*0.008* (36)	−**0.0006**	*0.002* (10)
	Contact zone I				
	*clavatus*‐like	−**0.012**	*0.025* (152)	**0.0008**	*0.009* (80)
	Intermediate	−**0.14**	*0.063* (157)	−**0.017**	*0.018* (56)
	*rubicundus*‐like	−**0.006**	*0.011* (103)	−**0.001**	*0.008* (70)
	Contact zone II				
	*clavatus*‐like	−**0.012**	*0.021* (14)	**0.002**	*0.005* (8)
	Intermediate	−**0.16**	*0.11* (19)	−**0.004**	*0.02* (7)
	*rubicundus*‐like	−**0.0005**	*0.008* (46)	−**0.001**	*0.009* (24)
	Contact zone III				
	*clavatus*‐like	−**0.01**	*0.016* (61)	−**0.0006**	*0.009* (32)
	Intermediate	−**0.22**	*0.12* (50)	−**0.02**	*0.023* (29)
	allop. *rubicundus*	−**0.002**	*0.007* (43)	−**0.004**	*0.006* (24)

For each contact zone, the estimates are given separately for the parental‐like and intermediate populations. Sample sizes are shown in parentheses. The values in italics are variances, the values on gray background are covariances, and the values in bold are estimates of LD. Asterisks indicate covariances showing high and significant correlations were found (see text). Δ*z*‐values are the maximum trait differences across the hybrid zone.

To exclude pleiotropy, we calculated covariance between the traits separately in allopatric and hybrid populations. As covariance in pure sites was not higher than in hybrid sites, pleiotropy seems unlikely. Therefore, we suggest that the increase in covariance in the centers of the contact zones is the result of high LD between quantitative phenotypic traits. Table 3 lists the estimates of LD between wings and antennae for males and females. As with covariance, LD was found to be highest in the sites with intermediate phenotypes. In CZI, LD for females was higher in *rubicundus*‐like than *clavatus*‐like populations, which contrasted with the covariance data. The relative values of LD and covariance in allopatric populations were also noncoincident: both sexes showed higher covariance in *clavatus* sites and higher LD in *rubicundus* sites. Meanwhile, LD was generally low in all allopatric populations.

## Discussion

### Structure of the hybrid zone revealed by a GIS approach

The published literature concerning the integration of GIS and hybrid zone research is not very extensive (Kohlmann et al. 1988; Kidd and Ritchie 2000; Jones and Searle 2003; Tauleigne‐Gomes and Lefébvre 2005; Swenson 2006, 2008). However, hybrid zone GIS research can clarify the structure and maintenance of a hybrid zone, as well as the dynamics of a hybrid zone over time. In this study, we visualize the hybrid zone between *S. clavatus* and *S. rubicundus* using IDW interpolation. This technique can effectively spread a frequency of phenotypes over previously unvisited sites that were not studied because of various technical reasons. Here, we were confronted with two technical difficulties: the inaccessibility of particular mountain slopes for investigators and difficulties in measurements of some phenotypic characters, namely in courtship song recordings. For example, spatial gaps between the most eastern and the main parts of CZI or between CZII and CZIII (Figs. 5A, 8A) resulted from the difficulties in collecting material on steep slopes. Meanwhile, the phenotype distributions across these contact zones give evidence that the gaps are actually smaller and may be overcome by grasshoppers.

We found three different contact zones between *S. clavatus* and *S. rubicundus* on a relatively small territory of Mount Tomaros. How can such a complex distribution of phenotypes on Mount Tomaros be explained? We assume that this distribution may be a result of a cyclic migration of populations during glacial and interglacial periods (Hewitt 1996). When ascending Mount Tomaros during an interglacial period, populations of *S. clavatus* and *S. rubicundus* could occasionally split into subpopulations of different size, with a relatively small number of *S*. *clavatus* and a high number of *S. rubicundus* in the northeastern part of Mount Tomaros, but the opposite distribution in its northern part. If individual characters were moved by drift, we expect a tendency for genes to flow predominantly from common to rare species (Dowling et al. 1989; Taylor and Hebert 1993; Wayne 1993). This, however, is not the case because in all contact zones, the wing clines are displaced toward the *rubicundus* end for both sexes, whereas the antenna clines are shifted to the *clavatus* end for males. Thus, this difference seems to be driven by selection rather than by drift.

Another explanation of the difference in the distributions of phenotypes between the three contact zones may be a variation in habitats across the contact zones. It is suggested that an ecotone can determine the position of a hybrid zone under exogenous selection. For example, Yanchukov et al. (2006) argued that habitat distribution and habitat preference can jointly affect the structure of hybrid zones in European *Bombina* toads. In the North American ground crickets (*Allonemobius*), a patchy distribution of local climatic conditions in a mountain hybrid zone leads to a similarly patchy distribution of genotypes (Howard 1986; Howard and Waring 1991). A tight correlation between soil type and genotype has also been shown in the mosaic hybrid zone between two North American cricket species of *Gryllus* (Ross and Harrison 2002). In the hybrid zone between *S. clavatus* and *S. rubicundus*, the cline difference may also be due to the joint effect of habitat distribution and habitat preference. *S. clavatus* prefers stony places, whereas *S. rubicundus* more likely occurs in grassy habitats (Sradnick 2010). However, in the mosaic hybrid zone between *Ch. brunneus* and *Ch. jacobsi,* the habitat variation explained only a small amount of the phenotypic variation (Bridle and Butlin 2002). Similarly, in the center of the *clavatus*/*rubicundus* hybrid zone, the distribution of phenotypes was unimodal at some sites and bimodal at others (Figs. 5A, 8A, 10A), which is difficult to explain based on habitat preferences. Because we also have the shift of the wing clines and the antenna clines to the opposite sides of each contact zone, habitat‐dependent selection seems to be unlikely. However, no detailed study of the habitat distribution on Mount Tomaros has been performed, nor have ecological preferences in hybrids been studied. This question calls for further investigation.

### Differences in cline width and position between the characters

The comparison of cline width and position reveals discordant and noncoincident clines for wings and antennae in CZI, the best studied zone on Mount Tomaros. In males, the wing cline is slightly wider than the antenna cline (although both clines are relatively narrow), and the wing cline is displaced more to the *rubicundus* end, whereas the antenna cline is diplaced toward the *clavatus* end of the contact zone (see Table 2, Figs. 5B, 8B). In females, the antenna cline is much wider than the wing cline. In CZII and CZIII, the clines for wings and antennae behave similarly, although the cline differences in widths and central placements are nonsignificant. Because all male characters and female wings show abrupt transitions across the contact zones, this cline difference seems to be driven by selection rather than by drift. Moreover, because both wings and antennae are actively used during courtship behavior, we suggest that sexual selection is the main driving force that keeps the zones narrow. How, then, can the cline shifts for the morphological characters in different directions be explained?

The courtship song of the Greek specimens of *S. rubicundus* typically consists of three phases, two of which incorporate wing movements (Elsner and Wasser 1995; Vedenina et al. 2012). Phase II of the courtship song includes sound pulses produced by two different mechanisms, leg movements and wing movements: low‐amplitude pulses produced by the legs alternate with high‐amplitude pulses produced by wing‐beats. Phase III of the courtship song is composed of only the wing stridulation. During the courtship of *S. rubicundus*, females are capable of seeing very conspicuous wing movements. Therefore, an acoustic component (song) of the courtship of *S. rubicundus* is enhanced by a visual display (wing movements), which may play a role in a female's choice of a male. By contrast, males of *S. clavatus* do not produce any wing movements during courtship, using only the leg stridulation that is more common for gomphocerine grasshoppers. There is an obvious correlation in differences between the species in behavior and wing morphology: the wings of *S. rubicundus* are wider and darker than the wings of *S. clavatus*, and the fields between the costal, subcostal, and medial veins of the hind wings are heavily sclerotized in both sexes of *S. rubicundus* (Harz 1975; Elsner and Wasser 1995) but not in *S. clavatus* (Willemse 1979). Displacement of the wing clines to the *rubicundus* end of the contact zone may indicate a higher attractiveness of *rubicundus* than *clavatus* wing phenotypes for females of both species. On the other hand, not only the male cline but also the female cline is displaced to the *rubicundus* end of the contact zone. We suggest two possible explanations for this result. First, the wing stridulation may be used by both sexes of *S. rubicundus* during long‐distance communication. It is known that the male calling song in *S. rubicundus* includes an element produced by wing vibration (Ragge and Reynolds 1998), and we documented that a receptive female sometimes responded using the same mechanism (J. Sradnick, pers. observ.). During long‐distance communication in gomphocerine grasshoppers, a male and a receptive female often sing a duet and move toward each other between song bouts (von Helversen and von Helversen 1983). Grasshopper females prefer to approach males that sing more loudly (von Helversen and von Helversen 1994); thus, we suppose that the reverse situation may be similar, that is, that males prefer females that respond more loudly. Sound pulses produced by wing‐beats are on average louder than the pulses produced by leg stridulation. Thus, on Mount Tomaros, the males may more actively move toward those females that produce a song by wing‐beats and therefore the wing characters in both sexes may be subject to sexual selection. A second explanation of the cline displacement to *rubicundus* side of the contact zone may be different dispersal capabilities of the parental phenotypes. Individuals of *S. rubicundus* show a stronger ability to fly than those of *S. clavatus*, which means a stronger dispersal of *S. rubicundus* than *S. clavatus*. Thus, the displacement of the wing clines across the hybrid zone may also be maintained by natural selection.

The male antennae show an abrupt transition across CZI; in females, by contrast, the antenna cline is much wider (Fig. 9A). The noncoincidence could occur because this trait is subject to different selection pressure in males and females. During a particular phase of the courtship song (phase III), *S. clavatus* males show elaborate movements of the spatulate antennae to attract the female's visual attention (Ostrowski et al. 2009). By contrast, *S. clavatus* females do not produce any antenna movements, nor do *S. rubicundus* males produce antenna movements during courtship. The displacement of the male antenna cline to the *clavatus* side of CZI may indicate a higher favorability of *clavatus* than *rubicundus* antenna phenotypes for females of both species. If evolution of female preferences is driven by sexual selection, an additional visual display that exaggerates courtship may be attractive for females. Various selection pressures acting on both sexes may also result from certain differences in sensory organs, which may help in the search for a potential mate. Enlarged apical segments of antenna may bear a higher number of chemoreceptors, which would be favorable for males in the search for females. However, as has been shown by Dumas et al. (2010) in distantly related gomphocerine species with club‐shaped antennae, such antennae are characterized by a lower number of olfactory or taste sensilla than are filiform antennae. By contrast, the club‐shaped antennae bear the higher number of mechanoreceptors. These authors explain the latter phenomenon by a possible involvement of the mechanoreceptors in the control of antenna movements because all grasshopper species with the spatulate antennae demonstrate conspicuous antenna movements during courtship. Thus, the male antennae on Mount Tomaros are most likely subject to sexual selection via female choice.

The song cline does not differ significantly from the wing cline across CZI either in width or position. This means that the song cline is displaced more to the *rubicundus* side than to the *clavatus* side of the contact zone. Such a result does not coincide with the analyses of inheritance pattern and pattern of female preferences of the courtship songs in *S. clavatus* and *S. rubicundus* (Vedenina et al. 2012, 2013). First, the *clavatus*‐like song elements were found to dominate in hybrid songs. Second, the females from intermediate populations of Mount Tomaros and the females of laboratory F1 hybrids also preferred the *S. clavatus* song over other song types. These results suggest a displacement of the hybrid zone in favor of *S. clavatus*. The behavioral experiments, however, were conducted by a playback of the courtship songs to females. This method may cause the overestimation of the degree of female responsiveness: a female does not receive any cues except acoustic ones, whereas during normal courtship, the female receives other stimuli (visual, chemical, tactile) as well. To clarify this, other methods should be used in behavioral experiments; in addition, more courtship songs should be recorded from more sites on Mount Tomaros to study the clinal song variation on a finer scale.

A difference in width between the male clines implies that selection is stronger on alleles at antenna loci than on alleles at wing and song loci. This means either that the same amount of selection is acting across more loci for wing morphology and song score or that selection on these characters is weaker than selection on antenna shape. The latter explanation is unlikely considering the approximately equal involvement of all three characters in reproductive success in these grasshopper species. By contrast, the difference in width between the female clines may substantially result from the differences in levels of selection. This puzzle could be resolved by an estimation of the number of loci affecting these traits. For example, small numbers of genetic factors have been shown to underlie the differences in most calling song characters and in stridulatory peg number between hybridizing grasshopper species *Ch. brunneus* and *Ch. jacobsi* (Saldamando et al. 2005). The width of the cline for song characters was found to be narrower than that for peg number (Bridle and Butlin 2002). Considering the inheritance pattern, the authors imply that selection on peg number is weaker overall than selection on song score. In the hybrid zone between *Ch. albomarginatus* and *Ch. oschei*, clines in most courtship song characters were found to be concordant and coincident (Vedenina 2011). However, a different pattern of inheritance was found for different song characters, which assumes polygenic control for some traits and few loci affecting other traits (Vedenina et al. 2007). Because all song characters seem to be the subject of strong sexual selection, the same selection is likely acting across a different number of loci for each character.

### Possible causes of changes in variance and covariance across the hybrid zone

For both morphological traits, the variances are found to increase in the centers of the contact zones. The highest covariance between the two morphological traits is also found in the sites with intermediate phenotypes. This, however, is much more strongly expressed in males than in females, and high and significant correlations between wings and antennae were only found in males. Because covariance in pure sites is not higher than in hybrid sites, pleiotropy seems unlikely, and an increase in covariance in the centers of the contact zones is the result of the high levels of LD. There are several possible causes of high levels of LD at the cline center. First, high LD could be generated if parental phenotypes move directly into the center of the hybrid zone. This explanation is consistent with the relatively small area of distribution of *S. rubicundus* and *S. clavatus* on Mount Tomaros and the dispersal capacity of these species. This capacity is, however, asymmetric: the dispersal of *S. rubicundus* should be stronger than that of *S. clavatus*. Second, strong assortative mating between parental phenotypes could also explain high levels of LD at the cline center. Playback of the courtship songs revealed assortative preferences in females of both species: they significantly prefer the songs of conspecific males (Vedenina et al. 2013). Third, higher levels of LD in the center than in the periphery of the hybrid zone may also suggest selection against hybrids. We presume that mainly extrinsic (exogenous) selection contributes to the increase in LD at the cline center; intrinsic (endogenous) selection seems to be unimportant. Our previous data demonstrate that genetic incompatibility between *S. rubicundus* and *S. clavatus* is relatively weak, whereas hybrid unfitness is expressed as a reduced mating success of hybrid males (Vedenina et al. 2012, 2013). Additionally, a consequence of hybrid inferiority driven by the partial incompatibility of the parental genomes is that the clines for different characters should be coincident and concordant (Barton and Gale 1993). However, in the *rubicundus*/*clavatus* hybrid zone, the clines for three characters are different. Thus, we suggest that all three factors mentioned above (renewed contact between parental phenotypes, strong assortative mating, and exogenous selection against hybrids) may contribute to the highly inflated levels of LD in the centers of all contact zones of Mount Tomaros for males. The modestly increased levels of LD that were found for females in CZI and CZIII may be explained either by renewed contact between parental phenotypes or by assortative mating between parental phenotypes.

The levels of covariance and LD are observed to be higher in *clavatus*‐like than *rubicundus*‐like populations of Mount Tomaros. Such asymmetry may be explained by a stronger assortative mating in the *clavatus‐*like sites than in the *rubicundus*‐like sites. In the behavioral experiments with playback of the courtship songs, females from *clavatus*‐like populations were more selective than females from *rubicundus*‐like populations, demonstrating significant preferences for *clavatus* songs (Vedenina et al. 2013). By contrast, females from *rubicundus*‐like localities did not demonstrate significant differences in response frequency between *rubicundus* and *clavatus* songs. Additionally, females obtained from backcrosses with *S*. *rubicundus* showed higher selectivity (responded more often to *rubicundus* songs) than females from *rubicundus*‐like populations of Mount Tomaros. This may be an indication that backcrosses to *S. rubicundus* do not form the major part of *rubicundus*‐like populations on Mount Tomaros but, instead, occur there together with other genotypes. Although these data are obtained on the basis of the courtship song preferences, they are consistent with the analysis of covariance between the two morphological traits. This supports our hypothesis that assortative mating is based on all three characters and that all male traits seem to be driven either by assortative mating or by selection against hybrids, with the contribution of drift being unlikely.

Asymmetry in mate discrimination found in *S. clavatus* and *S. rubicundus* may contribute to the future replacement of *S. rubicundus* by *S. clavatus* on Mount Tomaros (e.g., Shapiro 1998, 2001). In this hybrid zone that is classified as an “island” zone, the border of the range of *S. rubicundus* cannot shift indefinitely because this species does not occur below 1000 m a.s.l. (Berger et al. 2010). Therefore, *S. rubicundus* might become extinct on Mount Tomaros. Another scenario could be a spatial stabilizing of moving clines at an environmental boundary, simply because none of the hybridizing species would be capable of descending from Mount Tomaros.

## Conflict of Interest

The authors have no conflict of interests to declare.

## Supporting information


**Table S1.** Sampling sites on Mount Tomaros.
