# Supplementary material for: Variation in complex mating signals in an “island” hybrid zone between Stenobothrus grasshopper species
Source: Ecol Evol. 2016 Jun 26;6(14):5057–75. doi: 10.1002/ece3.2265 (PMC4979727; doi:10.1002/ece3.2265)
Supplement: Supplementary file 1 — Table S1. Sampling sites on Mount Tomaros. [file ECE3-6-5057-s001.doc]

**Table S1.** Sampling sites on Mount Tomaros.

| Name of locality | Geographical coordinates | Height (m) | Number of specimens (wing measurements) | Number of specimens (antenna measurements) |
| --- | --- | --- | --- | --- |
| Contact zone I | | | | |
| NR2 | 39°29'40,6"N 20°47'13,6"E | 1636 | 7 | 4 |
| NR3 | 39°29'35,0"N 20°47'16,0"E | 1717 | 2 | 2 |
| NR4 | 39°29'29,8"N 20°47'12,9"E | 1761 | 8 | 4 |
| NRM4 | 39°29'22,6"N 20°47'11,3"E | 1811 | 14 | 7 |
| N4 | 39°29'22,9"N 20°46'54,4"E | 1673 | 4 |  |
| TOPCAR | 39°29'32,1"N 20°46'49,8"E | 1564 | 7 |  |
| LIN | 39°29'30,3"N 20°46'46,7"E | 1555 | 34 |  |
| LI2 | 39°29'27,7"N 20°46'42,1"E | 1569 | 10 |  |
| LI1 | 39°29'17,7"N 20°46'47,2"E | 1661 | 26 | 13 |
| LCU | 39°29'30,5"N 20°46'36,9"E | 1545 | 51 | 17 |
| LIW1 | 39°29'16,7"N 20°46'42,4"E | 1621 | 14 | 11 |
| LIC1 | 39°29'27,3"N 20°46'37,3"E | 1581 | 14 |  |
| LIH | 39°29'28,3"N 20°46'30,1"E | 1543 | 19 | 15 |
| GRGIP | 39°29'16,4"N 20°47'30,3"E | 1918 | 10 |  |
| N5 | 39°29'15,8"N 20°47'03,8"E | 1757 | 18 |  |
| NS | 39°29'12,6"N 20°47'01,1"E | 1745 | 3 |  |
| NS2 | 39°29'11,4"N 20°47'11,5"E | 1778 | 6 | 4 |
| WH12 | 39°29'10,1"N 20°46'53,9"E | 1751 | 16 | 11 |
| WH11 | 39°29'07,6"N 20°46'52,0"E | 1741 | 12 | 9 |
| LIC | 39°29'28,5"N 20°46'36,2"E | 1568 | 19 |  |
| LHS | 39°29'26,6"N 20°46'27,8"E | 1536 | 28 | 15 |
| LIN2 | 39°29'21,5"N 20°46'40,3"E | 1631 | 18 |  |
| SARW5 | 39°29'10,6"N 20°47'23,7"E | 1884 | 44 | 16 |
| H1 | 39°29'10,2"N 20°47'20,9"E | 1854 | 45 | 12 |
| N8 | 39°29'08,4"N 20°47'07,5"E | 1780 | 16 |  |
| HZ5 | 39°29'08,3"N 20°47'26,6"E | 1888 | 29 | 18 |
| HYBRID | 39°29'06,6"N 20°47'10,8"E | 1790 | 30 | 19 |
| HYBGUT | 39°29'09,2"N 20°47'18,4"E | 1833 | 89 | 24 |
| S2 | 39°29'06,5"N 20°47'12,4"E | 1792 | 3 |  |
| SR6 | 39°29'06,1"N 20°47'29,2"E | 1885 | 49 | 24 |
| WH10 | 39°29'05,8"N 20°46'43,2"E | 1710 | 24 | 17 |
| N7 | 39°29'05,8"N 20°47'01,5"E | 1787 | 15 |  |
| WH3 | 39°29'05,7"N 20°46'33,7"E | 1617 | 8 | 7 |
| WH9 | 39°29'05,4"N 20°46'46,7"E | 1736 | 16 | 15 |
| N6 | 39°29'04,7"N 20°47'07,3"E | 1793 | 57 | 13 |
| WH8 | 39°29'04,3"N 20°46'50,0"E | 1761 | 10 | 9 |
| LF | 39°29'03,6"N 20°46'55,7"E | 1783 | 14 |  |
| WH7 | 39°29'02,1"N 20°46'52,5"E | 1784 | 42 | 19 |
| LIGIP | 39°29'00,3"N 20°46'56,4"E | 1803 | 110 | 25 |
| LC1 | 39°29'25,4"N 20°46'26,7"E | 1536 | 22 | 8 |
| LIH1 | 39°29'24,5"N 20°46'28,7"E | 1555 | 10 |  |
| MIX | 39°29'03,0"N 20°47'33,1"E | 1880 | 22 | 14 |
| 4TH | 39°29'00,2"N 20°47'36,8"E | 1876 | 28 | 8 |
| LIH2 | 39°29'20,3"N 20°46'29,4"E | 1581 | 17 | 16 |
| WH6O | 39°29'01,2"N 20°46'48,9"E | 1768 | 21 | 15 |
| WH6 | 39°28'59,3"N 20°46'46,0"E | 1750 | 18 | 15 |
| S3 | 39°29'01,6"N 20°47'03,5"E | 1792 | 23 | 14 |
| SATTEL | 39°28'59,5"N 20°47'01,6"E | 1786 | 1 |  |
| ZWSAM | 39°28'53,6"N 20°47'02,5"E | 1715 | 2 |  |
| G2 | 39°28'58,9"N 20°46'59,4"E | 1795 | 35 | 11 |
| G1 | 39°28'58,9"N 20°47'01,3"E | 1785 | 13 |  |
| RAST | 39°28'56,7"N 20°47'33,1"E | 1812 | 16 | 15 |
| OH5 | 39°29'27,6"N 20°47'58,8"E | 1425 | 16 | 5 |
| OHW | 39°29'18,7"N 20°48'05,9"E | 1405 | 23 | 16 |
| OH4-5 | 39°29'15,3"N 20°48'11,3"E | 1377 | 24 | 15 |
| OHN | 39°29'13,0"N 20°48'14,8"E | 1360 | 2 |  |
| OH4 | 39°29'09,2"N 20°48'18,1"E | 1358 | 27 | 9 |
| OH3-4 | 39°29'05,2"N 20°48'23,3"E | 1338 | 23 | 11 |
| OH3 | 39°29'03,4"N 20°48'26,2"E | 1321 | 54 | 9 |
| OH2 | 39°28'59,3"N 20°48'30,8"E | 1294 | 9 | 8 |
| ON0 | 39°28'57,3"N 20°48'34,0"E | 1291 | 9 |  |
| ON1 | 39°28'55,7"N 20°48'34,3"E | 1313 | 11 |  |
| ONB | 39°28'55,3"N 20°48'24,2"E | 1403 | 14 |  |
| ONA | 39°28'53,0"N 20°48'26,3"E | 1406 | 20 |  |
| ON2 | 39°28'52,1"N 20°48'35,1"E | 1345 | 11 |  |
| ON8 | 39°28'51,9"N 20°48'11,9"E | 1576 | 9 |  |
| ON7 | 39°28'51,9"N 20°48'14,6"E | 1547 | 15 |  |
| ON6 | 39°28'51,9"N 20°48'19,5"E | 1497 | 15 |  |
| ON5 | 39°28'50,1"N 20°48'24,2"E | 1455 | 11 |  |
| ON4 | 39°28'48,9"N 20°48'28,3"E | 1422 | 12 |  |
| ON3 | 39°28'47,9"N 20°48'36,2"E | 1379 | 16 |  |
| UR | 39°28'53,2"N 20°47'29,7"E | 1753 | 13 | 12 |
| OP | 39°28'48,7"N 20°47'23,6"E | 1644 | 13 | 11 |
| LIAL3 | 39°28'47,8"N 20°46'59,0"E | 1611 | 24 | 15 |
| LIAL2 | 39°28'43,7"N 20°47'01,3"E | 1543 | 10 | 8 |
| 3RD | 39°28'41,6"N 20°47'12,5"E | 1484 | 10 |  |
| SH | 39°28'40,4"N 20°46'43,0"E | 1471 | 8 |  |
| ALM1 | 39°28'38,9"N 20°47'17,8"E | 1514 | 85 | 12 |
| LIAL1 | 39°28'37,7"N 20°47'02,1"E | 1476 | 18 | 13 |
| 2ND | 39°28'35,0"N 20°47'09,4"E | 1425 | 36 | 15 |
| 1ST | 39°28'27,7"N 20°47'05,5"E | 1368 | 43 | 10 |
| START | 39°28'23,3"N 20°47'05,6"E | 1326 | 31 | 10 |
| Contact zone II | | | | |
| N1F | 39°30'40,1"N 20°47'11,7"E | 1365 | 11 |  |
| N1D | 39°30'36,2"N 20°47'24,1"E | 1378 | 13 | 9 |
| N1C | 39°30'35,2"N 20°47'23,3"E | 1398 | 43 | 18 |
| N1L | 39°30'33,3"N 20°47'13,1"E | 1392 | 30 | 7 |
| N1E | 39°30'29,7"N 20°47'24,9"E | 1445 | 8 |  |
| N1 | 39°30'27,3"N 20°47'03,6"E | 1438 | 47 | 15 |
| N1G | 39°30'25,0"N 20°47'34,6"E | 1489 | 10 | 8 |
| N1H | 39°30'24,9"N 20°47'39,7"E | 1468 | 12 | 7 |
| HP1 | 39°30'18,9"N 20°47'35,1"E | 1591 | 9 | 7 |
| N2L | 39°30'17,3"N 20°47'29,6"E | 1637 | 31 | 15 |
| HP2 | 39°30'14,5"N 20°47'40,4"E | 1401 | 25 | 8 |
| N2G | 39°30'12,3"N 20°47'38,3"E | 1630 | 20 |  |
| N2F | 39°30'09,5"N 20°47'39,2"E | 1627 | 12 |  |
| N2E | 39°30'06,1"N 20°47'38,9"E | 1618 | 17 |  |
| N2B | 39°30'05,2"N 20°47'27,4"E | 1619 | 13 |  |
| N2A | 39°30'03,7"N 20°47'19,7"E | 1595 | 11 |  |
| N2C | 39°30'03,6"N 20°47'32,4"E | 1653 | 26 |  |
| N2D | 39°30'02,4"N 20°47'37,2"E | 1649 | 13 |  |
| N2 | 39°29'57,6"N 20°47'08,6"E | 1524 | 37 |  |
| NR1 | 39°29'49,1"N 20°47'12,5"E | 1578 | 20 |  |
| Contact zone III | | | | |
| NTA | 39°32'43,2"N 20°44'47,7"E | 1267 | 18 |  |
| NT1 | 39°32'35,2"N 20°44'53,6"E | 1281 | 41 | 9 |
| NTB | 39°32'25,8"N 20°44'44,6"E | 1330 | 14 |  |
| NTC | 39°32'17,5"N 20°44'52,8"E | 1381 | 19 |  |
| NT2 | 39°32'07,6"N 20°44'56,1"E | 1419 | 12 | 9 |
| NTE | 39°32'00,3"N 20°45'07,5"E | 1453 | 15 |  |
| NTF | 39°31'54,1"N 20°45'28,7"E | 1456 | 14 |  |
| NT3 | 39°31'43,4"N 20°45'26,9"E | 1516 | 22 | 9 |
| NTH | 39°31'34,0"N 20°45'23,2"E | 1505 | 12 |  |
| NTI | 39°31'25,4"N 20°45'31,8"E | 1554 | 20 |  |
| NT4 | 39°31'22,6"N 20°45'54,8"E | 1621 | 2 | 3 |
| NT6 | 39°31'13,5"N 20°46'03,0"E | 1720 | 19 | 12 |
| NT5 | 39°31'12,1"N 20°45'57,4"E | 1667 | 29 | 13 |
| NTD | 39°31'11,7"N 20°46'12,5"E | 1757 | 19 | 15 |
| NT7 | 39°31'11,5"N 20°46'09,4"E | 1769 | 18 | 9 |
| NT8 | 39°31'09,6"N 20°46'14,3"E | 1747 | 11 | 8 |
| NT9 | 39°31'03,6"N 20°46'20,4"E | 1775 | 17 | 8 |
| NTG | 39°31'02,8"N 20°46'28,7"E | 1765 | 53 | 19 |
| A1 | 39°31'00,6"N 20°46'07,1"E | 1704 | 8 |  |
| NT11 | 39°30'57,6"N 20°46'20,0"E | 1776 | 10 | 8 |
| NT16 | 39°30'57,2"N 20°46'27,0"E | 1759 | 15 | 13 |
| NT15 | 39°30'56,6"N 20°46'22,8"E | 1759 | 11 | 11 |
| NT12 | 39°30'54,3"N 20°46'16,3"E | 1761 | 12 | 8 |
| NT13 | 39°30'52,7"N 20°46'12,4"E | 1738 | 8 | 8 |
| NT14 | 39°30'52,2"N 20°46'05,2"E | 1684 | 9 | 8 |
| NTS3 | 39°30'39,6"N 20°45'48,0"E | 1650 | 7 | 2 |
| NTS4 | 39°30'38,9"N 20°45'57,3"E | 1689 | 18 | 8 |
| NTS2 | 39°30'36,4"N 20°45'35,0"E | 1603 | 9 | 4 |
| NTS1 | 39°30'30,7"N 20°45'31,3"E | 1540 | 15 | 5 |
